# Examining the mediating roles of self-esteem and social support in the link between digital addiction and mental health: findings from a meta-analytic structural equation modeling study

**DOI:** 10.3389/fpubh.2026.1812488

**Published:** 2026-05-29

**Authors:** Turgut Karaköse, Tijen Tülübaş, Bünyamin Han, Sedat Kanadli, Abdurrahman Kardas, Hakan Polat, Murat Demirkol

**Affiliations:** 1 Faculty of Education, Kütahya Dumlupinar University, Kütahya, Türkiye; 2 Faculty of Education, Mersin University, Mersin, Türkiye; 3 Provincial Directorate of National Education, Batman, Türkiye; 4 Faculty of Education, Firat University, Elazig, Türkiye

**Keywords:** adolescents, digital addiction, mental health, self-esteem, social support, university students

## Abstract

**Introduction:**

In their constantly digitalizing environments, adolescents and university students become more vulnerable to digital addiction and related mental health problems. Existing evidence has shown that perceived social support and self-esteem are significant psycho-social factors influencing their tendency to experience digital addiction and mental health problems. However, studies investigating the interrelationships between these constructs are limited in number despite their growing significance for this cohort of individuals.

**Methods:**

This study aims to test a proposed research model using meta-analytic structural equation modeling (MASEM) analysis in order to reveal the multifaceted relationships between these significant constructs. The study particularly addresses the mediating role of perceived social support and self-esteem in the relationship between digital addiction and mental health. Analyses were made by using correlation coefficients retrieved from a total of 182 studies accumulating a total sample of 111.798 participants.

**Results:**

The results showed that digital addiction of late-adolescents and university students was statistically associated with their mental health both directly and indirectly through perceived social support and self-esteem.

**Discussion:**

By testing an integrated model of psychological and behavioral factors within a single exploratory framework, the study provided an original contribution to the literature and suggested significant implications for incentives to be designed not only to support young people's effective use of digital technologies but to protect their mental health through strengthening their social ties and self-esteem.

## 1 Introduction

The rapid advancements in internet-based and mobile technologies, artificial intelligence, several other forms of digital media have profoundly transformed contemporary social life (98, 101). The ongoing digital transformation has particularly reshaped the daily routines, social relationships, learning styles, and educational processes of young adults and students (81), which has not only provided benefits but also brought various risks to the well-being of individuals. One of the most prominent concerns is the ever-increasing risk of digital addiction resulting from the excessive, uncontrolled, and compulsive use of digital technologies (95). Digital addiction, which is conceptualized as a complex process shaped by the interactions between identity development, attachment styles, and emotional regulation mechanisms (99), has indeed become a growing social problem (1) and been linked to a range of psychological and behavioral challenges (100, 106).

Excessive exposure to and problematic use of digital technologies have become some of the most debated topics in the field of mental health due to their numerous negative consequences (80, 92, 111). Research indicates that digital addiction can disrupt daily life activities, weaken impulse control, and lead to significant impairment in social relationships (89, 93). Empirical studies consistently demonstrate strong associations between digital addiction and various psychological risks, including depression, anxiety, stress, loneliness, low self-esteem, attention problems, and sleep disorders (94, 96, 108).

Evidence also shows that young people are most prone to the negative effects of problematic technology use and are more likely to exhibit symptoms of digital addiction (78, 95, 102). Studies report high prevalence of digital addiction among university students (83, 84, 103, 105), which might have been compounded by the widespread use of online education systems in higher education and the increasing demand for digital communication among university students (82, 104). Several factors have been identified as triggers for digital addiction in young people, including the search for social belonging (103), academic stress and anxiety (86), the search for social approval and pressures to present oneself (85), increased leisure time, constant access to personal digital devices (104), and the impact of digital platforms on identity formation processes (107).

It is widely accepted that digital addiction is multifacetedly related to mental health problems. For example, social media addiction, a subtype of digital addiction characterized by a constant desire to stay online, has been shown to increase social comparison and consequently trigger depressive symptoms (79, 112). Similarly, intensive smartphone use has been associated with increased levels of stress and difficulties in emotion regulation (91, 110). Sleep disturbance is another critical consequence because prolonged exposure to screens late at night suppresses melatonin secretion, leading to reduced sleep duration and decreased sleep quality (87, 97). Research also suggests that the relationship between digital addiction and mental health problems is bidirectional. Digital addiction increases depression, anxiety, and stress, and these mental health problems reinforce individuals' tendency to seek escape on digital platforms, leading them to perpetuate addictive behaviors (109, 113). Therefore, growing evidence shows that the impact of digital addiction on mental health occurs in a complex, multifaceted, and reciprocal cycle (23).

However, not all individuals with high levels of exposure to digital media experience mental health problems, which indicates the significant mediating role of psychosocial factors in the addiction-mental health relationship. Among these factors, social support and self-esteem are widely recognized as two key psychological resources that might play a central role. Strong perceived social support has been shown to protect individuals from psychological distress and enhance overall well-being (2, 3). While the online environment itself is not inherently addictive, inadequate social support resulting from deficiencies in social and family relationships can increase vulnerability to digital addiction (4, 5). Conversely, social support received from close social networks can reduce anxiety symptoms in adolescents and mitigate the negative effects of social media addiction (6). Systematic reviews supporting this perspective have consistently shown significant associations between social support and mental health outcomes (7).

Self-esteem is another factor closely linked to mental health due to its central role in adolescent psychological problems (8) and association with psychiatric conditions during adolescence (90). Beyond its direct relationship with mental health, self-esteem constitutes a significant psychological resource that facilitates adaptive coping, and an increasing number of studies show that self-esteem is significantly influenced by perceived social support (9). Therefore, low self-esteem can increase vulnerability to emotional distress and can reinforce maladaptive patterns of digital use. Empirical evidence shows that individuals with low self-esteem are more prone to social media addiction (10).

While previous studies have examined the relationship between digital addiction, mental health problems, social support, and self-esteem separately or bidirectionally, research addressing the mediating roles of social support and self-esteem in the digital addiction-mental health relationship is limited. Addressing this void in the literature, this study aims to investigate the relationship between digital addiction and mental health problems of late-adolescents and university students and explore the mediating effects of social support and self-esteem. By conducting a cumulative assessment of existing results in the literature within an integrated model, the study provides more generalizable and comprehensive results that can enhance our understanding of the multiple relationships between these significant constructs and guide the development of effective interventions aiming to promote the psychological well-being of young people and encourage their healthier engagement with digital technologies.

## 2 Theoretical background and hypothesis building

### 2.1 Digital addiction and mental health problems

Despite their numerous benefits, excessive use of digital media such as smartphones or social media can also lead to significant problems (11, 15) and poses a risk of addiction for young people (12–14). A high correlation has been reported between problematic digital technology use and various psychological problems such as depression, anxiety, and sleep disorders (11, 15–20). Research has shown that problematic internet use may be associated with various psychopathological symptoms (21, 22). For instance, young people with digital addiction reported low self-health ratings (23), and increased smartphone use and smartphone addiction led to a lower quality of life and mental health problems (24). With a particular emphasis on university students, Zhao et al. (25) reported that digital addiction had a negative impact on their physical and mental health. These findings suggest that digital addiction can lead to mental health problems in the context of university students and late adolescents, and thus, we propose the following hypothesis:

*H1. Digital addiction has a significant positive effect on mental health problems*.

### 2.2 Social support and digital addiction

Empirical studies have shown that perceived social support has a significant effect on digital addiction, with higher levels of support associated with lower addictive behaviors (3, 26). Specifically, real-life social support has been found to reduce young people's excessive use of social media, thus lowering their likelihood of developing addictive patterns (2, 27). Among adolescents, strong negative associations have been reported between social support and smartphone/internet addiction, suggesting that supportive interpersonal relationships function as a protective factor against excessive digital media use (28, 29). On the other hand, young people who lack adequate social support from friends or family were found to be more prone to smartphone addiction (4, 5).

Longitudinal evidence also shows a unidirectional causal relationship from social support to internet addiction over time, highlighting the lasting impact of supportive networks in reducing addictive digital behaviors (30). Research focusing on university students also confirms a negative relationship between perceived social support and social media addiction (31). Family-based support can be particularly effective. For instance, young people who spend more time with their mothers reported higher perceived social support and lower internet use (32). Beyond behavioral consequences, social support has been shown to protect young adults' mental health from the negative effects of social media use (6). Recent studies also underscore social support as a protective factor against the risk of digital addiction by reducing vulnerability to excessive and maladaptive technology use (33). Taken together, we propose the following hypothesis:

*H2. Social support has a negative relationship with digital addiction*.

### 2.3 Self-esteem and digital addiction

Self-esteem is another significant psychological variable that is consistently associated with various forms of digital addiction. Studies among adolescents show a strong negative correlation between smartphone addiction and self-esteem, suggesting that individuals with low self-esteem are more prone to problematic technology use (28). Similarly, social media use has been identified as a significant predictor of self-esteem and its associated positive and negative outcomes (34). Empirical findings also reveal that individuals with low self-esteem are more likely to develop Facebook addiction, particularly in late adulthood, compared to those with high self-esteem (10). Additionally, self-esteem has been shown to negatively and significantly predict nomophobia among students, strengthening its protective role against digital addiction (35). Similarly, previous studies also show a significant correlation between self-esteem and the intensity of social media use (36). Based on this existing evidence, we propose the following hypothesis:

*H3. Self-esteem has a negative relationship with digital addiction*.

### 2.4 Social support and mental health problems

Social support has been identified as a critical factor in protecting mental health, particularly during adolescence and young adulthood. Support from significant individuals such as teachers, parents, and close friends can help young people develop a sense of purpose in life, leading to a more positive outlook and psychological resilience (37). Conversely, individuals experiencing higher levels of negative emotional states tend to report lower perceived social support from family members and significant others, highlighting the close interplay between emotional well-being and social resources (38). Both perceived and actual forms of social support can reduce vulnerability to mental disorders and help protect mental health (26). Emerging evidence suggests that perceived social support is associated with improved outcomes in serious mental health conditions, including schizophrenia, bipolar disorder, and anxiety disorders (39). It has also been shown that increased social support acts as a protective shield against mental health problems in young people (40).

Furthermore, systematic reviews provide robust evidence of a strong relationship between social support and mental health, emphasizing that higher levels of social support are associated with better psychological outcomes and recovery processes, while insufficient social support is associated with increased mental health problems and health inequalities (7). Collectively, these findings lead us to propose the following hypothesis:

*H4. Social support has a negative relationship with mental health problems*.

### 2.5 Self-esteem and mental health problems

The current literature emphasizes the central role of self-esteem in protecting adolescents' and young adults' mental health. While individuals may be aware of stigmatizing societal representations of mental illness, those who can reject negative labels threatening their sense of self exhibit more adaptive responses to mental health stigma, highlighting the critical role of self-esteem in this process (41). Empirical evidence shows that adolescents with psychiatric disorders report significantly lower levels of self-esteem compared to healthy control groups (42, 90). Low self-esteem has been identified as a significant explanatory factor for adolescents' internalization problems (8) and has been associated with worse mental health outcomes, particularly during early adolescence (43). Furthermore, it has been shown that labeling oneself as mentally ill is associated with decreased self-esteem, while abandoning these labels is associated with improved self-esteem (44), suggesting a dynamic relationship between self-esteem and psychological well-being. Consistent with this view, higher self-esteem has been found to play a central role in reducing mental health problems among adolescents (45). Taken together, these findings lead us to the following hypothesis:

*H5. Self-esteem has a negative relationship with mental health problems*.

### 2.6 Social support and self-esteem

Reciprocal peer support plays a critical role in boosting self-esteem, particularly among individuals experiencing mental health challenges (46). Studies with adolescents and young adults show that higher levels of perceived social support are associated with higher self-esteem (47, 48). This relationship is well explained by attachment theory and social support models, which emphasize the role of supportive interpersonal relationships in the development of positive self-evaluations (49). From a theoretical perspective, theories of self-esteem development suggest that self-esteem emerges through social interactions, perceived competence, and feedback from significant others across developmental stages (50).

Evidence suggests that social support affects self-esteem both directly and indirectly. While Goodwin et al. (51) found that self-esteem is indirectly influenced by perceived social support, longitudinal findings show that self-esteem also plays a determinant role in the development of adolescents' social support networks, highlighting a reciprocal and reinforcing relationship (52, 53).

The protective function of social support and self-esteem is also evident in regulating mental health outcomes. Low self-esteem, when combined with inadequate social support, significantly increases the risk of post-stress depression (54), whereas self-esteem functions as a key mechanism linking social support to a reduction in suicidal ideation (55). In higher education contexts, social support and self-esteem collectively contribute to students' psychological and academic adjustment (56) and enhance well-being and happiness by promoting positive emotions (57). Recent neuroscientific findings further strengthen this relationship, showing that perceived social support can influence self-esteem through underlying neuroanatomical mechanisms (9). Based on this existing evidence, we propose the following hypothesis:

*H6. Social support has a positive relationship with self-esteem*.

### 2.7 Social support mediating the relationship between digital addiction and mental health problems

The protective role of social support against various types of digital addiction and related mental health issues is emphasized in the current literature. Previous studies have shown that social support can decrease digital addiction by enhancing individuals' well-being and reducing the risk of mental health problems (3). Furthermore, social support has been indirectly associated with internet addiction, demonstrating its role in shaping individuals' technology use behaviors through mediating psychological processes (58). Empirical evidence also shows that social support can have a significant negative effect on young people's internet addiction (59), associating average or higher levels of perceived social support with reduced social media use and fewer general, physical, and psychological health symptoms (2). Furthermore, social support, particularly support from family members and close social networks, has been found to reduce anxiety symptoms in adolescents as well as mitigate the negative impact of social media addiction on anxiety severity (6). Negative emotional states are well-known risk factors for technological addictions, as individuals' excessive gaming or social media use can be a sign of maladaptive coping strategies used against these negative emotions (60). Therefore, perceived social support can play a buffering role by mitigating the impact of negative emotional states on addictive digital behaviors (38) and promoting psychological resilience, particularly among university students (61). Taken together, these findings suggest that social support may not only independently reduce digital addiction and mental health problems but also function as a fundamental mechanism linking the two. Therefore, we propose the following hypothesis:

*H7. Social support mediates the relationship between digital addiction and mental health problems*.

### 2.8 Self-esteem mediating the relationship between digital addiction and mental health problems

Self-esteem plays a central role in explaining individuals' vulnerability to digital addiction and its psychological consequences. Empirical findings indicate that individuals with low self-esteem are more prone to social media addiction, and those with high levels of narcissism show increased addictive tendencies, highlighting the importance of self-related constructs in problematic digital media use (10). Furthermore, previous studies have shown that self-esteem mediates the relationship between the intensity of social media use and addictive or compulsive behaviors, and that low self-esteem increases susceptibility to negative consequences associated with overuse (36). Evidence also shows that social media use among university students is significantly associated with depressive symptoms (62, 63), suggesting that digital addiction is closely linked to mental health symptoms. Similarly, low self-esteem is commonly observed in adolescents with psychiatric disorders (42) and plays a significant role in the emergence of psychopathology related to peer aggression and identity development during adolescence (8). As suggested by Sevelko et al. (64), low self-esteem can have a central role in both digital addiction and mental health problems.

Adolescents experiencing higher levels of emotional distress tend to report more social media addiction, which predicts low self-esteem (65). Consistent with these findings, depression, anxiety, and interpersonal sensitivity have been closely associated with internet addiction, while low self-esteem is often linked to problematic internet use among students (66). Taken together, these results lead us to promote the following hypothesis:

*H8. Self-esteem mediates the relationship between digital addiction and mental health problems*.

### 2.9 The proposed research model

**Figure 1:**
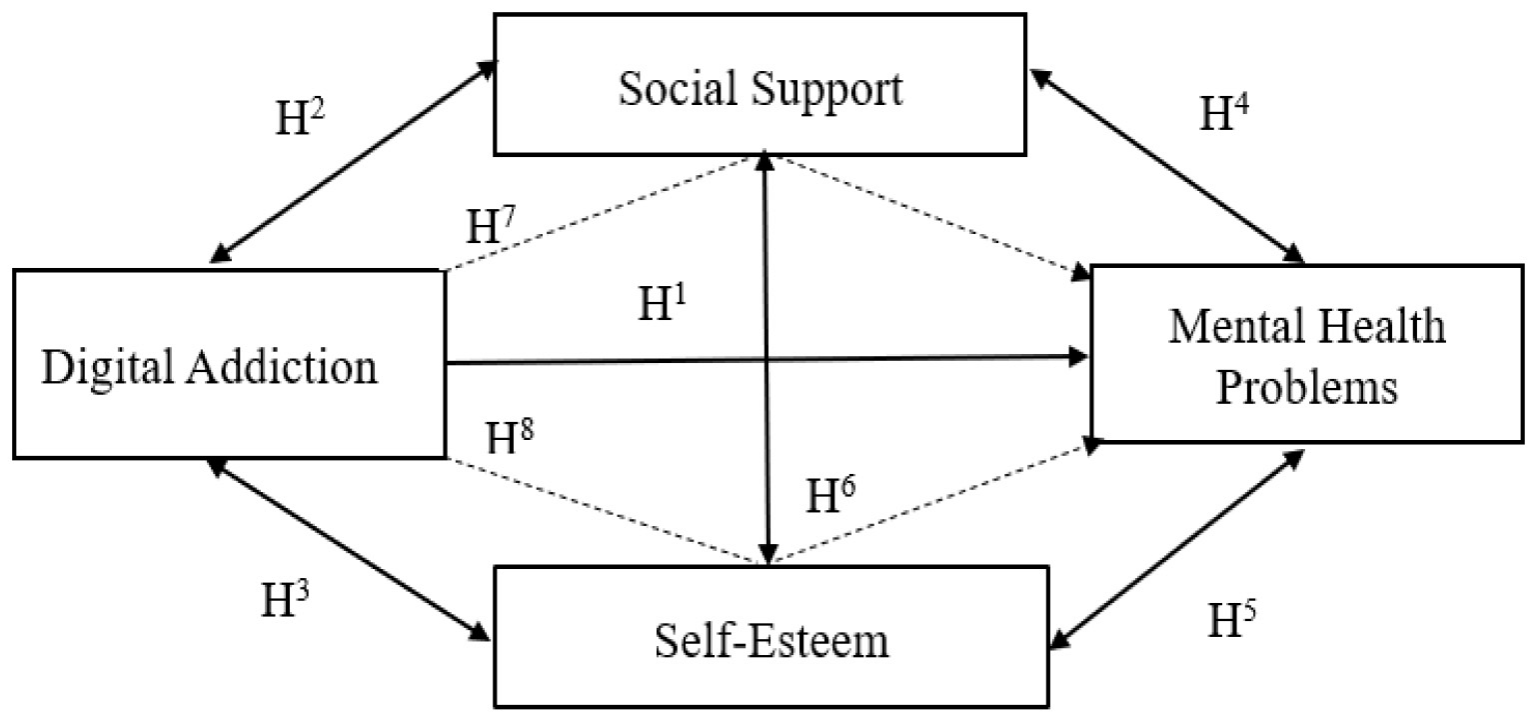
The proposed research model.

Using the eight hypotheses developed over the existing evidence regarding the relationships between study variables, we constructed the research model presented in Figure 1.

## 3 Methodology

### 3.1 Data collection

Data for this study were collected from Web of Science (WoS), Scopus, and Google Scholar. The initial search was performed on 17 December 2025, simultaneously on WoS and Scopus, using combinations of keywords relevant to digital addiction, including smartphone, social media, and video game addiction, mental health, social support, and self-esteem. As the study targeted university students and late-adolescents, this was also included in the search string. With this initial search, a total of 218 documents were retrieved from WoS and 274 records from Scopus. At this stage, 186 documents were extracted from the list due to being duplicates, so a total of 306 documents remained for detailed screening.

In accordance with the PRISMA 2020 guidelines (67), a comprehensive data extraction process was subsequently conducted, as illustrated in Figure 1.

**Figure 2:**
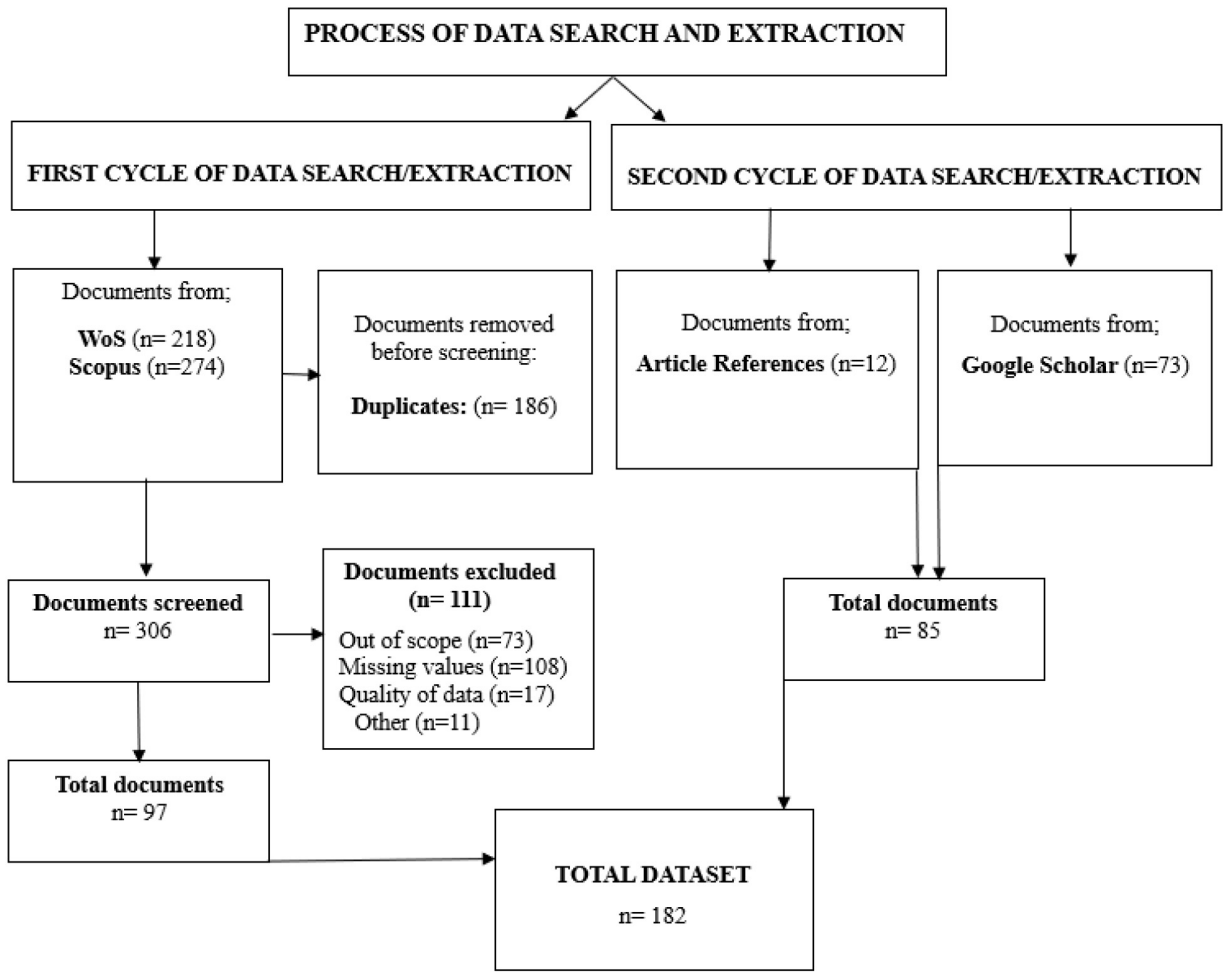
The PRISMA-2020 flowchart for data collection and extraction.

As shown in Figure 2, we first submitted 306 documents from the initial search to detailed screening to evaluate whether they provided eligible data for analysis. MASEM analyses are performed using the Pearson correlation values reported by primary documents. Therefore, this was our primary criterion for including/excluding documents. Out of 306 documents, 73 was excluded for being out of scope (e.g. about early or middle adolescence, elementary or lower-secondary school context), 108 were excluded for not providing correlation values, 17 were excluded for not providing quality data (e.g. data not rigorously/clearly presented, too small sampling or insufficient information about the sample), and 11 were excluded for other reasons (e.g., language or unavailable full text). At this stage, the references of these articles were screened to detect any documents that our first search might have missed, and 12 documents were detected to be included in the analyses. Next, we conducted a second cycle of search on Google Scholar to identify any new or recent documents that can be included in our analysis, which provided an additional 73 documents. Finally, a total of 182 documents were included in the final dataset.

### 3.2 Data analysis

The mediating role of social support and self-esteem in the relationship between digital addiction and mental health problems was tested using the 182 correlation effect sizes collected from the relevant literature. The goodness-of-fit of the proposed model and three alternative models was tested using these effect sizes. For this purpose, the two-stage structural equation modeling-TSSEM method (68, 69) was used. In the first stage, the correlation effect sizes were combined using the random effects model (70) to calculate average effect sizes. The scale proposed by Funder & Ozer (88) for behavioral sciences was used to interpret the calculated effect sizes. According to this scale, correlation effect sizes up to 0.10 are interpreted as “very small”, up to 0.20 as “small”, up to 0.30 as “medium”, up to 0.40 as “wide”, and 0.40 and above as “very wide”. A heterogeneity test was also conducted to determine the presence and magnitude of variance between studies. The significant *Q* value calculated with this test (*p* < 0.05) indicates the presence of variance between studies, which stems from the characteristics of the studies, excluding sampling error. The magnitude of this variance is determined by the *I*^2^ percentage value. Although the *I*^2^ value is not absolute, up to 25% is interpreted as “low”, up to 50% as “medium”, and up to 75% as “high” heterogeneity (71).

In the second stage of the TSSEM, the goodness-of-fit of the proposed model and alternative models was calculated using the covariance matrices created in the first stage. For this purpose, the RMSEA, SRMR, CFI, and TLI indices were examined. An RMSEA value equal to or <0.05 was considered an “excellent” fit, and a value between 0.05 and 0.08 was considered an acceptable fit. An SRMR value equal to or <0.08 was considered an excellent “fit,” whereas a value between 0.08 and 0.1 was considered an acceptable fit (72–74). For comparative fit indices, TLI and CFI values equal to or >0.95 were interpreted as “excellent fit”, while values between 0.90 and 0.95 were interpreted as “acceptable fit” (72, 73). In addition, the AIC and BIC values were used as criteria to determine the most suitable model for the data, and the model with lower values in both of these was interpreted to have the best data fit (75). Data analyses were performed using the web-based webMASEM developed by Jak et al. (76).

## 4 Results

### 4.1 Summary effects

The correlation coefficients collected from the studies included in the meta-analysis (*N* = 111,798) were combined using the random effects model to create correlation matrices. The correlation effect sizes obtained are given in Table 1.

**Table 1:** Summary of the results obtained from the application of the MASEM procedure.

Associations	*k*	Total sample	*r*	%95 Confidence Interval		*I^2^*
				Lower limit	Upper limit	
DA&MH	91	70909	0.351	0.322	0.380	%93.9
DA&SS	37	23958	−0.303	−0.362	−0.244	%95.9
DA&SE	21	11824	−0.360	−0.428	−0.292	%95
MH&SS	49	29059	−0.363	−0.406	−0.321	%95.4
MH&SE	38	19039	−0.497	−0.541	−0.452	%95.2
SS&SE	68	38358	0.265	0.190	0.340	%98.5

DA, digital addiction; MH, mental health problems; SS, social support; SE, self-esteem.

As can be seen in Table 1, the correlation effect size between digital addiction and mental health problems was calculated as *r* = 0.351, 95% CI [0.322, 0.380], which represents a “large” effect size. Accordingly, an increase in participants' digital addiction leads to an increase in their mental health.

The correlation effect size between digital addiction and social support was calculated as *r* = −0.303, 95% CI [−0.362, −0.244], and the correlation effect size between digital addiction and self-esteem was calculated as *r* = −0.360, 95% CI [−0.428, −0.292]. These results suggest that digital addiction and social support is negatively and moderately correlated while there is a “large” negative correlation between digital addiction and self-esteem. Accordingly, higher levels of digital addiction were associated with higher levels of mental health problems.

The correlation effect size between social support and mental health problems was calculated as *r* = −0.363, 95% CI [−0.406, −0.321], and between self-esteem and mental health as *r* = −0.497, 95% CI [−0.541, −0.452]. These results suggest that both social support and self-esteem had a “large” negative correlation with mental health problems, indicating that participants' with higher levels of social support and self-esteem exhibited lower levels of mental health problems. Finally, a “moderate” and significant correlation effect size [*r* = 0.265, 95% CI (0.190, 0.340)] was determined between participants' social support and self-esteem scores, which indicates that participants' with higher levels of perceived social support demonstrated higher levels of self-esteem.

Table 1 shows that *I*^2^ values range from 93.9% to 98.5%, which indicates a “high” level of variance between effect sizes, suggesting that a significant portion of the variation in effect sizes may stem from the specific characteristics of the studies included in the meta-analysis.

### 4.2 Test of the proposed model

In the second stage of meta-analytic structural equation modeling, the correlation matrices obtained were combined to test the fit of the established model with the data. The goodness-of-fit indices obtained from the analysis are given in Table 2.

**Table 2:** Summary of the goodness-of-fit indices for the proposed model.

Model	χ2(df)	*p*-value	RMSEA	RMSEA %95 Li	RMSEA %95 Ui	SRMR	TLI	CFI	AIC	BIC
Original model	21.71(1)	0.000	0.014	0.009	0.019	0.054	0.919	0.987	19.71	10.01

**Figure 3:**
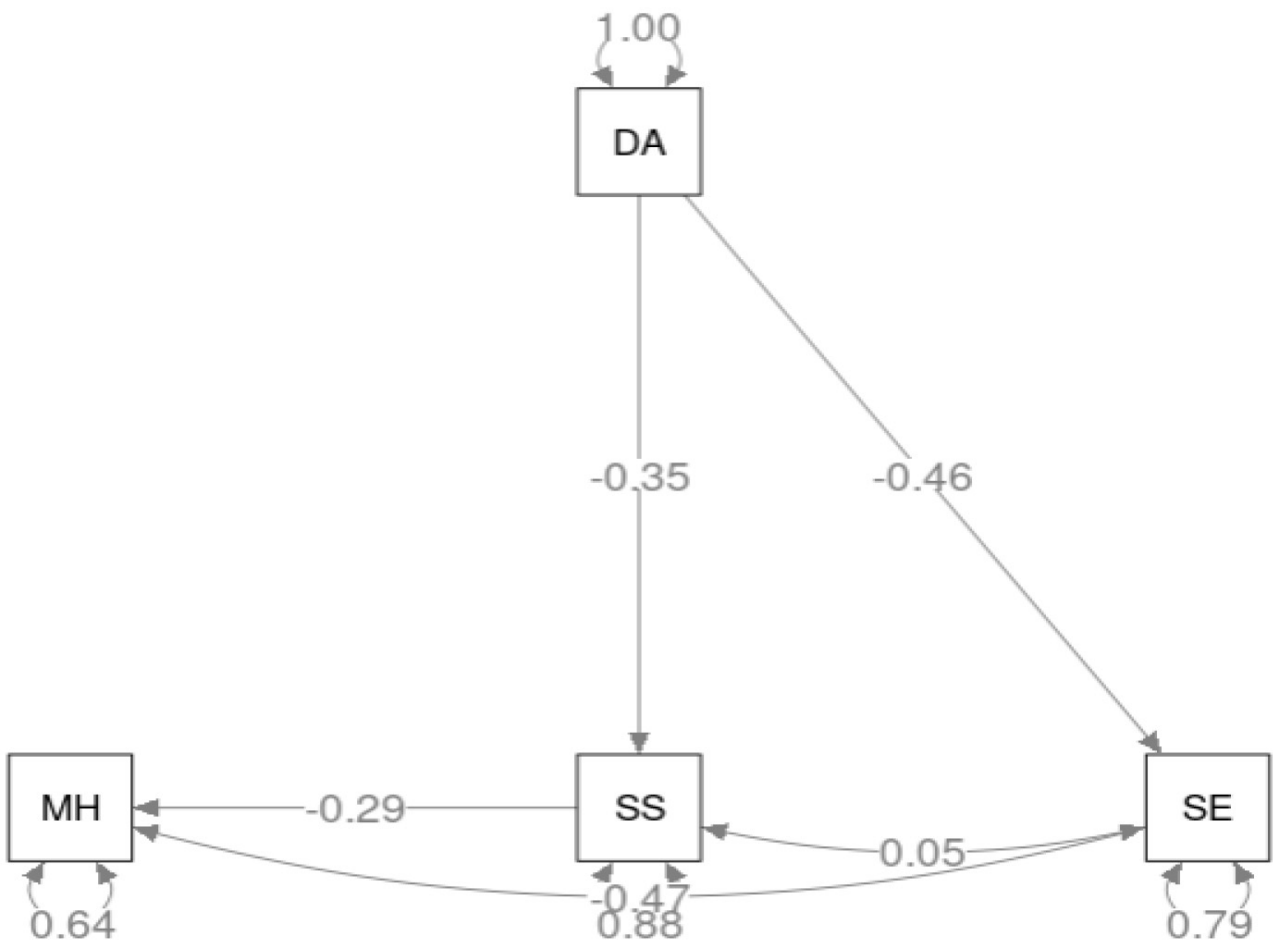
Path model of the proposed model with parameter estimates and 95% confidence intervals (DA, digital addiction; MH, mental health problems; SS, social support; SE, self-esteem).

As shown in Table 2, the chi-square test for one degree of freedom of the proposed model was not statistically significant (χ^2^ = 21.71, *p* < 0.05). RMSEA showed an “excellent” fit with a 95% CI of 0.014 [0.009, 0.019], SRMR showed an “acceptable” fit with a value of 0.054, TLI showed an “acceptable” fit with a value of 0.91, and CFI showed an “excellent” fit with a value of 0.987, suggesting that the model showed a good fit to the data. The path analysis diagram drawn for the proposed model is illustrated in Figure 3.

As can be seen in the path model (Figure 3), digital addiction had a “very large” negative effect on self-esteem [β = −0.457, 95% CI (−0.511, −0.403)] and a “large” negative effect on social support [β = −0.347, 95% CI [−0.403, −0.291)]. Digital addiction explained 12% of the variance in social support and 21% of the variance in self-esteem. These results indicate that digital addiction was significantly negatively associated with both social support and self-esteem in the structural model.

**Figure 4:**
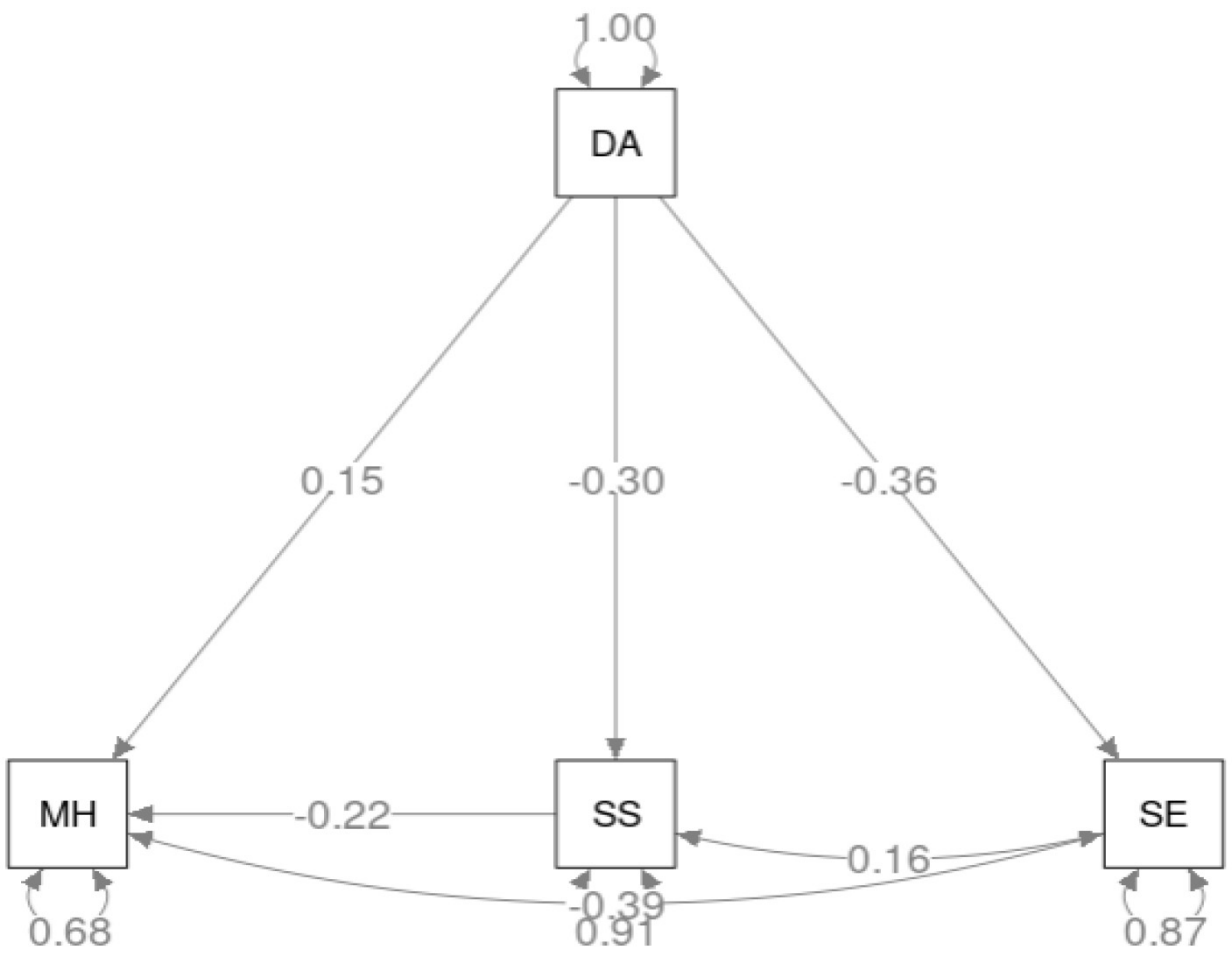
Path model of the mediation model with parameter estimates and 95% confidence intervals (DA, digital addiction; MH, mental health problems; SS, social support; SE, self-esteem).

According to the path model, social support had a “moderate” negative effect on mental health problems [β = −0.286, 95% CI (−0.341, −0.231)] while self-esteem had a “very large” negative effect [β = −0.473, 95% CI (−0.518, −0.428)]. Social support and self-esteem explain 36% of the variance in mental health problems indicating that higher levels of social support and self-esteem were significantly associated with lower mental health problems. The examination of the covariance relationship between social support and self-esteem defined in the model revealed a “very small” positive [*r* = −0.05, 95% CI (−0.021, −0.121)] but insignificant (*p* > 0.05) relationship. These findings indicate that social support and self-esteem significantly mediate the relationship between digital addiction and mental health problems. To determine whether these variables are full or partial mediators, a regression arrow was drawn from digital addiction (DA) to mental health problems (MH). The path graph of this mediating model is shown in Figure 4.

No goodness-of-fit index was calculated because the mediating model was saturated (df = 0). The effect of digital addiction on mental health problems was found to be positive, small, and significant [β = 0.147, 95% CI (0.093, 0.199)]. Accordingly, social support and self-esteem were partial mediators in the relationship between digital addiction and mental health problems. In other words, the effect of digital addiction on mental health occurred both directly and indirectly through social support and self-esteem, suggesting that the statistical association between digital addiction and mental health problems is partly accounted for by lower levels of social support and self-esteem.

### 4.3 Test of alternative models

In addition to the proposed model, the model-data fit of three alternative models was tested, and the results are presented in Table 3.

**Table 3:** Summary of the goodness-of-fit indices for the alternative models.

Model	χ2(df)	*p*-value	RMSEA	RMSEA %95 Li	RMSEA %95 Ui	SRMR	TLI	CFI	AIC	BIC
Model 1	90.10(2)	0.000	0.020	0.016	0.023	0.113	0.826	0.942	80.70	60.78
Model 2	272.57(2)	0.000	0.035	0.031	0.038	0.182	0.474	0.824	268.6	249.3
Model 3	270.40(2)	0.000	0.035	0.031	0.038	0.167	0.478	0.826	266.4	247.15

**Figure 5:**
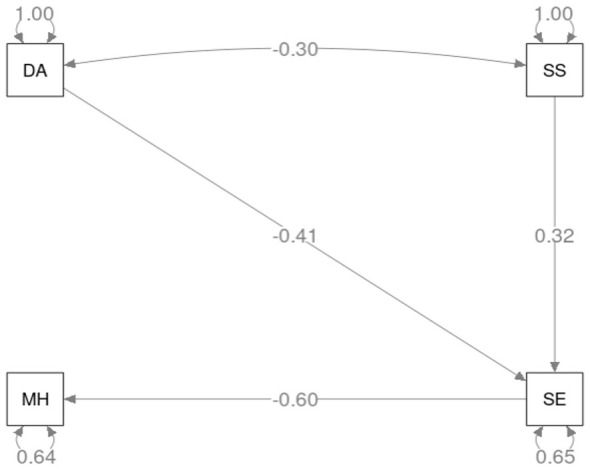
Path model of the Model 1 with parameter estimates and 95% confidence intervals (DA, digital addiction; MH, mental health problems; SS, social support; SE, self-esteem).

In the first alternative model (Model 1), digital addiction and social support were selected as independent variables, and both variables predicted mental health problems through self-esteem. The path graph of the model is given in Figure 5.

All regression coefficients of Model 1 were found to be significant at the 95% confidence level (*p* < 0.05). According to the goodness-of-fit indices for Model 1 (see Table 3), the RMSEA value of 0.020 95% [0.016, 0.023] showed an “excellent” fit, and the CFI value of 0.942 showed an “acceptable” fit. However, the SRMR (0.113) and TLI (0.826) indices were below the acceptable fit. Because a high degree of freedom (df = 2) adversely affects comparative fit indices such as TLI (74), this value may be considered low. When the fit indices are considered collectively, the model provides an acceptable fit to the data.

**Figure 6:**
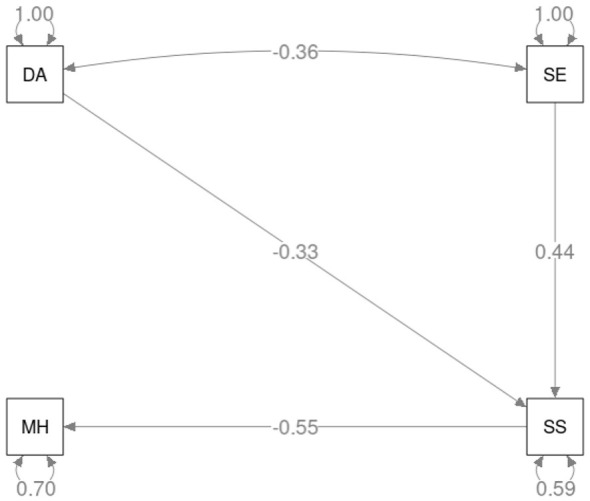
Path model of the Model 2 with parameter estimates and 95% confidence intervals (DA, digital addiction; MH, mental health problems; SS, social support; SE, self-esteem).

In Model 2, digital addiction and self-esteem were selected as independent variables, and both were determined as predictors of social support. Social support was found to be a predictor of mental health problems. The path graph of Model 2 is shown in Figure 6.

All regression coefficients of Model 2 were found to be significant at the 95% confidence level (*p* < 0.05). According to the goodness-of-fit indices (see Table 3), this model shows an excellent fit with an RMSEA value of 0.035, while SRMR (0.182), whereas the TLI (0.474) and CFI (0.824) values are below acceptable fit. Accordingly, it can be said that this model does not fit the data well.

**Figure 7:**
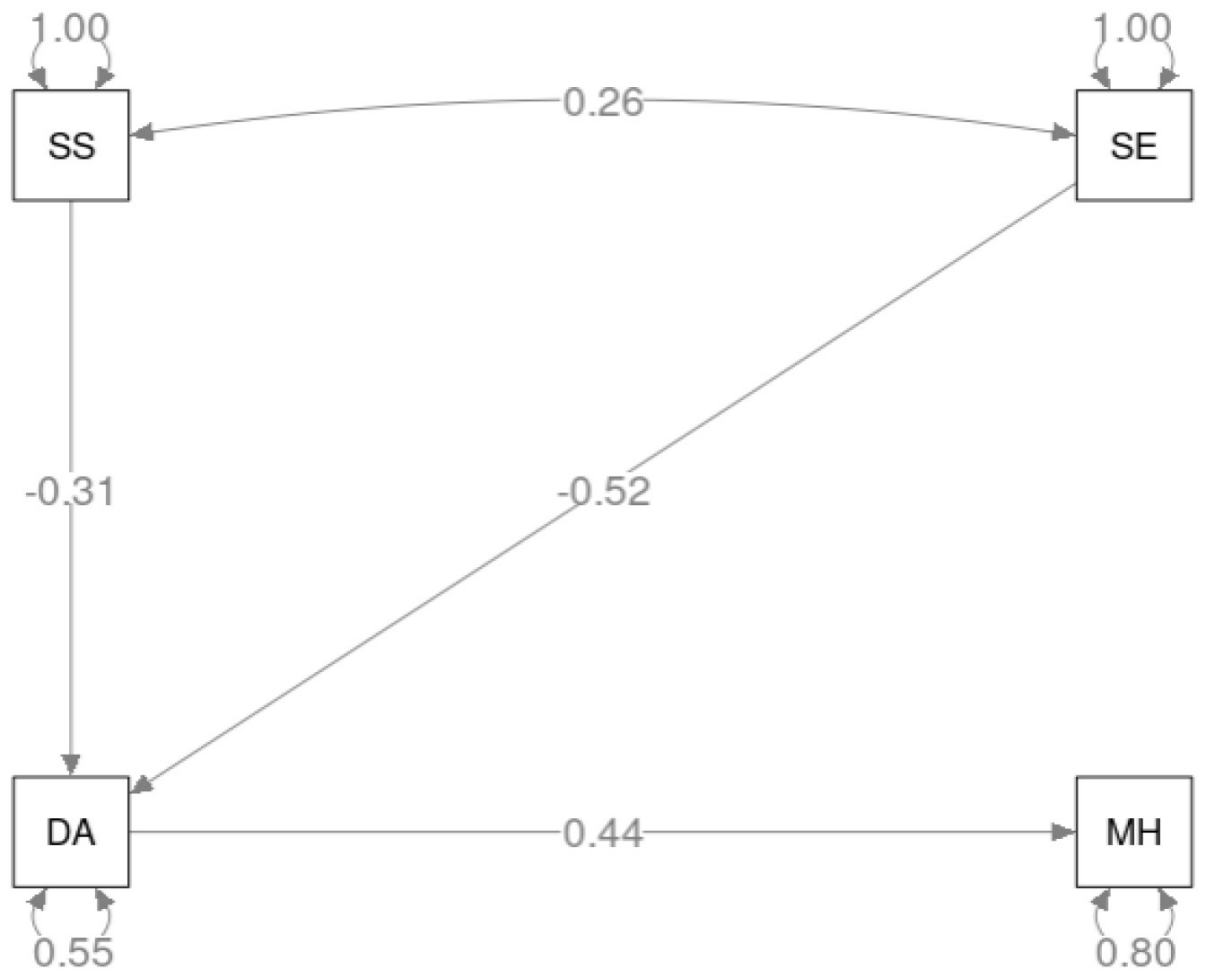
Path model of the model 3 with parameter estimates and 95% confidence intervals (DA, digital addiction; MH, mental health problems; SS, social support; SE, self-esteem).

In Model 3, social support and self-esteem were determined as independent variables, digital addiction as a mediating variable, and mental health problems as the dependent variable. The path graph of Model 3 is shown in Figure 7.

All regression coefficients of Model 3 were statistically significant at the 95% confidence level (*p* < 0.05). According to the goodness-of-fit indices given for this model in Table 3, RMSEA (0.035) showed excellent fit, but SRMR (0.167), TLI (0.478), and CFI (0.826) were below the acceptable values. Although there was an increase in TLI (0.798), it still remained below the acceptable limits, suggesting that Model 3 was insufficient in explaining the relational structure in the dataset, and therefore, model-data fit was not achieved.

To determine the most suitable model for the data among the models tested, the AIC and BIC indices were calculated. According to Kline (73), a model with lower AIC and BIC indices is considered more suitable for the data than a competing model. Accordingly, the AIC value for the Proposed Model was 19.71, and the BIC value was 10.01. These values were the lowest compared to the values of alternative models. Among the alternative models, Model 1 (AIC = 80.70 and BIC = 60.78), Model 2 (AIC = 268.6 and BIC = 249.3), and Model 3 (AIC = 266.4 and BIC = 247.15) were respectively more suitable for the data. Accordingly, considering the goodness-of-fit indices of the proposed model, it can be said that the model-data fit was optimal in the Proposed Model.

## 5 Discussion

This study tested the multiple relationships between late-adolescents and university students' mental health problems, digital addiction, perceived social support, and self-efficacy, with a particular focus on the mediating roles of social support and self-efficacy in the relationship between digital addiction and mental health. For this purpose, pairwise correlation effect sizes were calculated for the pairwise relationships between the variables using data from a total of 182 studies, corresponding to a sample size of 111.798 participants. Correlation coefficients were obtained from the literature, and correlation effect sizes were combined using a random-effects model. Accordingly, the correlation effect size between digital addiction and mental health problems was found to be significant, positive, and “wide” (*r* = 0.35, *p* < 0.05).

The analysis revealed a large positive correlation between digital addiction and mental health problems, which is consistent with the existing evidence in the literature. Earlier studies found that excessive and uncontrolled use of digital technologies posed psychological risks for young people (11, 15). Specifically, excessive use of social media or similar digital platforms resulted in addictive behaviors among students, such as compulsive use, loss of control, and functional impairment (12–14). Empirical studies consistently demonstrated a strong association between problematic use of digital technologies and numerous mental health problems, including depression, anxiety, and sleep disorders (11, 15–20). Similar patterns have been reported in previous research on the relationship between pathological internet use and various psychopathological symptoms (21, 22). Several mechanisms exist that could explain this relationship. Excessive digital interaction can disrupt sleep-wake cycles, reduce face-to-face social interaction, and increase exposure to social comparison and negative online content, all of which are known risk factors for poor psychological well-being. Furthermore, adolescents with digital addiction have been found to have lower self-assessments of their health status (23), suggesting that digital addiction may weaken individuals' perceptions of overall well-being. In parallel, increased smartphone use and higher levels of addiction have been associated with lower quality of life and greater mental health problems (24). Recent evidence also shows that digital addiction is negatively associated with both the physical and psychological health of college students (25). Taken together, digital addiction can be interpreted as a significant psychosocial risk factor exacerbating mental health problems in young people.

A moderate positive correlation was found between social support and digital addiction, suggesting that lower levels of perceived social support can lead to digital addiction or vice versa. Consistent with this finding, the literature underscores that higher levels of social support are associated with lower levels of digital addiction. An increasing number of empirical studies have shown that perceived social support has a direct negative impact on digital addiction, and individuals with stronger support networks exhibit lower levels of addictive behaviors (3, 26). Specifically, real-life social support has been shown to reduce young people's dependence on social media by meeting their social and emotional needs offline, thus decreasing their likelihood of developing addictive usage patterns (2, 27). Among adolescents, strong negative associations between social support and both smartphone and internet addiction suggest that supportive interpersonal relationships function as a protective factor against excessive digital interaction (28, 29). On the other hand, exposure to digital pressure or inadequate social support significantly increases vulnerability to smartphone addiction (4), while weak social and family relationships have been identified as significant contributing factors to internet addiction (5). Longitudinal evidence further supports a unidirectional causal relationship from social support to internet addiction, highlighting the protective role sustained over time by supportive networks (30). Similar patterns have been observed among university students, where perceived social support is negatively associated with social media addiction (31). Family-based support appears to be particularly effective, as increased maternal involvement is associated with higher perceived support and a weaker tendency to develop digital addiction (32). Beyond behavioral outcomes, social support has been shown to mitigate negative mental health effects of social media use and strengthen resilience to digital addiction, thus reinforcing its role as a significant protective factor against excessive and maladaptive technology use (6, 33).

This study also revealed a large, negative relationship between social support and mental health problems, suggesting that social support can decrease late-adolescents' and university students' likelihood to experience mental health problems, which adds to the existing evidence on the protective effect of social support on mental health, particularly during adolescence and young adulthood. Support from significant individuals, such as parents, teachers, and close friends, enhances a sense of purpose and meaning, which in turn increases positive outlook and psychological resilience (37). Conversely, individuals experiencing higher levels of negative emotional states tend to report lower perceived social support, highlighting the close and reciprocal relationship between emotional well-being and social resources (38). Both perceived and tangible forms of social support, including financial assistance and direct services, have been shown to reduce vulnerability to mental disorders and contribute to better mental health outcomes (26). Emerging evidence also shows that higher perceived social support is associated with improved outcomes, even in serious mental health conditions such as schizophrenia, bipolar disorder, and anxiety disorders (39). Increased social support among young people has been shown to protect against the development of mental health problems (40). Furthermore, systematic reviews provide robust evidence of a strong relationship between social support and mental health, showing that higher levels of support are associated with better psychological outcomes and recovery. In contrast, insufficient support is associated with increased mental health problems and health inequalities (7).

Self-esteem was found to have a large positive correlation with digital addiction, suggesting that higher levels of self-esteem is significantly linked to reduced levels of digital addiction. Previous findings lend support to this argument by identifying self-esteem as a significant psychological factor in reducing digital addiction. Studies among young people show a strong negative relationship between self-esteem and smartphone addiction, indicating that individuals with low self-esteem are more prone to problematic technology use (28). Similarly, social media use has been identified as a significant determinant affecting self-esteem and its associated positive and negative outcomes (34). Empirical evidence suggests that individuals with low self-esteem, especially in later developmental stages, are more likely to develop Facebook addiction compared to those with high self-esteem (10). Self-esteem has also been shown to significantly and negatively predict nomophobia among students, strengthening its protective role against digital addiction (35). These findings are further supported by evidence linking self-esteem to the intensity of social media use (36). In summary, high self-esteem can reduce the need for external validation, social comparison, and emotional compensation through digital platforms, thus lowering the risk of excessive and maladaptive digital interaction.

A very large negative correlation was determined between self-esteem and mental health problems, indicating the significant role of self-esteem in maintaining mental health. Early research supports this stance by highlighting self-esteem as a central protective factor in adolescent mental health. Adolescents who can resist stigmatizing labels related to mental illness and maintain a positive sense of self tend to respond more adaptively to mental health stigma, which underscores the critical role of self-esteem in psychological adjustment (41). Empirical evidence shows that adolescents with psychiatric disorders report significantly lower self-esteem compared to their healthy peers (42, 90), and low self-esteem has been identified as a significant explanatory factor for internalization problems in adolescence, particularly during early developmental stages (8, 43). Furthermore, it has been shown that labeling oneself as mentally ill is associated with decreased self-esteem, while abandoning these labels is associated with improved self-esteem, suggesting a dynamic interaction between self-perception and psychological well-being (44). From a theoretical perspective, theories of self-esteem development suggest that engaging in supportive and social behaviors increases self-esteem, which in turn supports overall well-being (50). In line with this view, higher self-esteem has consistently been shown to play a central role in reducing mental health problems among adolescents (45).

The other two variables included in the analyses, social support and self-esteem, was found to have moderate positive correlation, underlining the reciprocal influence between the constructs and pointing to the significant role of social support in facilitating the self-efficacy of young people. This is consistent with existing evidence on the strong positive relationship between social support and self-esteem. Studies with adolescents and young adults have consistently shown that higher levels of perceived social support are associated with higher self-esteem, highlighting the importance of supportive interpersonal relationships in the development of positive self-evaluations (47, 48). Consistent with attachment theory and social support models, receiving emotional validation, acceptance, and encouragement from significant others strengthens individuals' sense of self-worth and competence (49). Evidence also shows that social support affects self-esteem both directly and indirectly; perceived support increases self-esteem, while higher self-esteem facilitates the development and maintenance of supportive social networks, demonstrating a reciprocal and reinforcing relationship (51, 52). This relationship appears particularly strong during adolescence, as social support plays a central role in the formation of the self-concept (53). Furthermore, peer and family support have been shown to be particularly beneficial for individuals experiencing mental health issues by contributing to improvements in self-esteem and psychological adjustment (46). New evidence from educational and neuroscientific research further supports this link, demonstrating that social support and self-esteem collectively promote psychological well-being, resilience, and adaptive function through both psychosocial and neurobiological mechanisms (9, 56, 57).

Test of the original research model against three alternative models supported the proposed model relative to the alternatives. Accordingly, digital addiction was shown to have a large negative association with social support and a very large negative association with self-esteem, suggesting that digital addiction can be a significant determinant of both social support and self-esteem while it can also function not only as a consequence but also as a significant predictor of both social support and self-esteem. Consistent with previous research, excessive and maladaptive technology use can weaken real-life social interactions, thereby reducing perceived social support from family and peers (2, 59). As individuals increasingly rely on digital platforms to cope with negative emotional states, this shift can further erode supportive social networks and limit access to protective interpersonal resources (38). Similarly, the literature shows that digital addiction is closely linked to low self-esteem because excessive social media use intensifies social comparison, reliance on external validation, and sensitivity to negative feedback (10, 36). Over time, these processes can damage individuals' self-esteem and contribute to greater psychological vulnerability. This makes digital addiction a significant factor that can gradually decrease social support and self-esteem (64).

As proposed in the original model, both social support and self-esteem were found to have a moderate negative association with mental health problems, indicating that higher levels of perceived social support and self-esteem associated with lower levels of mental health problems in late-adolescents and university student. Consistent with this finding, previous research suggests that social support and self-esteem function together as complementary protective factors in reducing mental health problems. Social support directly reduces psychological distress by providing emotional security and coping resources, which can in turn mitigate the harmful effects of maladaptive digital behaviors (3, 6). In addition, self-esteem can strengthen individuals' internal resilience by promoting positive self-evaluations and reducing vulnerability to depressive and anxiety-related symptoms associated with digital addiction (10, 64). Higher levels of social support can make individuals feel valuable and accepted, which can promote their self-esteem and reduce their reliance on digital media for emotional compensation (36). Taken together, these resources appear to work synergistically where social support reduces external stressors and emotional tension and self-esteem facilitates healthier self-regulation and coping, thereby collectively contributing to lower levels of mental health problems.

The mediation analysis showed that both social support and self-esteem statistically mediate the relationship between digital addiction and mental health problems, underscoring that digital addiction is statistically associated with mental health problems both directly and indirectly through social support and self-esteem. It should be noted that the term “mediation” is used here in its statistical rather than causal sense (77), since the primary studies contributing to the pooled correlations are predominantly cross-sectional. In other words, the results suggest that higher levels of digital addiction are associated with lower perceived social support and self-esteem, which are in turn associated with greater mental health problems. Prior research showed that excessive and maladaptive use of digital media was directly related to higher psychological distress, partly because individuals used technology as an avoidance strategy in response to negative emotional states, which later intensified anxiety and depressive symptoms (10, 36, 38, 63). Similarly, digital addiction can erode real-life social relationships and lead to decreased perceived social support, which then mitigate the negative mental health effects of internet and social media addiction (2, 3). Since low self-esteem is a well-known risk factor for poor mental health, it appears to function as one of the key psychological pathways through which digital addiction contributes to mental health problems (64). Taken together, these findings support a bidirectional mechanism where digital addiction directly exacerbates psychological distress while simultaneously weakening vital protective psychosocial resources (i.e., social support and self-esteem) essential for mental health.

### 5.1 Limitations

The results of this study should be interpreted in light of several limitations inherent in meta-analytical analysis. First, MASEM requires the reporting of Pearson correlation coefficients among relevant variables; therefore, studies that did not provide sufficient statistical information could not be included, which may have reduced the comprehensiveness of the dataset. Second, variability across studies in terms of sample characteristics, cultural contexts, research designs, and measurement instruments may introduce heterogeneity that cannot be fully controlled, potentially affecting the stability and generalizability of the results. Third, MASEM relies on aggregated correlation matrices rather than individual-level data, limiting the ability to examine more nuanced relationships. However, the current analysis was strengthened by testing the fit of alternative models.

An important limitation is the substantial between-study heterogeneity, with *I*^2^ values between 93.9% and 98.5%. The two-stage SEM approach (69) accounts for this through random-effects pooling, but heterogeneity of this magnitude means the pooled correlations describe a distribution of effects rather than a single common effect. Our aim here was to compare the proposed model against alternative structural specifications, not to explain why effect sizes vary across studies, so study-level moderators were not coded *a priori*. The pooled estimates and the parameters of the best-fitting model should therefore be read as average tendencies across a heterogeneous literature. Therefore, future MASEM studies in this area would benefit from coding study and sample characteristics and carrying out subgroup analyses or meta-regression on the structural parameters.

## 6 Conclusion

This study moved beyond the existing studies in the literature by testing the multiple relationships between four significant constructs influencing the health functioning of teenagers and young adults in the contemporary fast-digitalizing world, namely digital addiction, social support, self-esteem, and mental health problems. By going beyond investigations into the bidirectional relationships between these variables in relatively small samples, the study provided a more comprehensive, multi-layered and generalizable results that can guide both theory and practice.

One significant finding was that both social support and self-esteem statistically mediated the association between digital addiction and the mental health of young individuals, such that higher levels of digital addiction were associated with lower perceived social support and self-esteem, which were in turn associated with poorer mental health. This adds up to the widely-acknowledged view that people with low self-esteem or those who seek social support often resort to digital media, which may be associated with digital addiction in the long run. Taken together, it can be stated that in the case of late-adolescents and university students, digital addiction is not only a risk factor that might weaken their social ties or self-esteem but can also result from their poor self-esteem or seek for social support. In both cases, perceived social support and self-esteem emerge as significant mechanisms, both singly and collectively, that regulate the influence of digital addiction on their mental health problems. Building on this evidence, it can be stated that efforts to promote young people's effective and responsible use of digital media are important but not sufficient on their own. It is equally critical to support adolescents in developing healthy social relationships and networks and to strengthen their self-esteem. Such protective factors can play a key role in alleviating the mental health problems associated with digital addiction.

## Data Availability

The original contributions presented in the study are included in the article/supplementary material, further inquiries can be directed to the corresponding author.
